# Rat as a Predictive Model for Human Clearance and Bioavailability of Monoclonal Antibodies

**DOI:** 10.3390/antib14010002

**Published:** 2024-12-24

**Authors:** Jason D. Robarge, Kevin M. Budge, Lucy Her, Andrea M. Patterson, Patricia Brown-Augsburger

**Affiliations:** Eli Lilly and Company, Lilly Corporate Center Indianapolis, Indianapolis, IN 46285, USA; jason.robarge@lilly.com (J.D.R.); budge_kevin@lilly.com (K.M.B.); her_lucy@lilly.com (L.H.); andrea.patterson@lilly.com (A.M.P.)

**Keywords:** monoclonal antibody, rat, bioavailability, clearance, allometric scaling, pharmacokinetics

## Abstract

Background: The prediction of human clearance (CL) and subcutaneous (SC) bioavailability is a critical aspect of monoclonal antibody (mAb) selection for clinical development. While monkeys are a well-accepted model for predicting human CL, other preclinical species have been less-thoroughly explored. Unlike CL, predicting the bioavailability of SC administered mAbs in humans remains challenging as contributing factors are not well understood, and preclinical models have not been systematically evaluated. Methods: Non-clinical and clinical pharmacokinetic (PK) parameters were mined from public and internal sources for rats, cynomolgus monkeys, and humans. Intravenous (IV) and SC PK was determined in Sprague Dawley rats for fourteen mAbs without existing PK data. Together, we obtained cross-species data for 25 mAbs to evaluate CL and SC bioavailability relationships among rats, monkeys, and humans. Results: Rat and monkey CL significantly correlated with human CL and supported the use of species-specific exponents for body-weight-based allometric scaling. Notably, rat SC bioavailability significantly correlated with human SC bioavailability, while monkey SC bioavailability did not. Bioavailability also correlated with clearance. Conclusions: The rat model enables an early assessment of mAb PK properties, allowing discrimination among molecules in the discovery pipeline and prediction of human PK. Importantly, rat SC bioavailability significantly correlated with human SC bioavailability, which has not been observed with other species. Rats are cost-effective and efficient relative to monkeys and provide a valuable tool for pharmacokinetic predictions in therapeutic antibody discovery.

## 1. Introduction

Monoclonal antibodies (mAbs) are an established class of biotherapeutics that are successfully used to treat diseases in many therapeutic areas. Monoclonal antibodies are also useful constructs that can form the backbone of next-generation time-extended biotherapeutics. Important features of optimal mAb therapeutics include high target specificity and affinity, slow clearance (CL), half-lives that can extend beyond one month, and subcutaneous (SC) bioavailability of up to 100% [1]. The therapeutic dosing intervals of mAbs with favorable pharmacokinetic (PK) and pharmacodynamic (PD) properties can be relatively infrequent, ranging from once weekly to quarterly [2]. However, PK properties of some mAbs can be suboptimal, necessitating higher and more frequent dosing, thereby limiting their therapeutic potential. Identifying predictive models to select or engineer mAbs with optimal human CL and SC bioavailability is an important goal in drug discovery.

Biophysical properties of mAbs that increase non-specific interactions with the in vivo environment may lead to faster CL, and in silico and in vitro strategies have been explored to identify predictors of PK characteristics to guide the selection or engineering of optimized molecules (as reviewed by [3,4]). For example, mAb isoelectric point (pI), cell surface binding to heparin, and binding to proteins such as baculovirus have been correlated with CL [3,5,6]. These tools have the benefit of high throughput and opportunity for early elimination of candidates predicted to have poor clearance. However, these tools have limitations as they can provide ambiguous or conflicting results, and do not fully recapitulate the array of complex in vivo interactions that determine clearance.

While various preclinical in vivo models have been explored to predict mAb PK in humans, monkeys are perhaps the most widely studied model. A common use of this animal model stems from high protein sequence homology and similarity in physiology with humans. Moreover, many antibody discovery and engineering strategies are directed at generating mAbs that are cross-reactive to human and monkey target proteins for the purpose of establishing a pharmacologically relevant study model. Monkey models are, therefore, often used to study target-dependent mAb disposition and are also recognized as good predictors of non-specific human CL [7,8]. The desirable characteristics of monkeys as preclinical models are offset by considerations, such as limited animal access, cost, study logistics, and need for skilled technicians. In view of the ethical impetus for overall reduction in the use of non-human primate (NHP), alternative models to predict the human PK properties of mAb therapeutics are highly desired.

Therapeutic mAb doses generally achieve circulating concentrations that saturate target binding sites [2]. At these doses, total mAb CL, which is the sum of target-dependent and non-specific CL, is predominated by non-specific clearance pathways. Major non-specific clearance mechanisms include non-specific pinocytosis by vascular endothelial cells or Fc-mediated endocytosis by phagocytic cells of the hepatic reticuloendothelial system, followed by either catabolism or recycling by the neonatal Fc receptor (FcRn) [7,8,9,10,11]. Given that non-specific CL is a primary determinant of total CL, non-clinical models do not have to be limited to those with target binding. Rodents are attractive alternatives to study non-specific mAb PK in drug discovery, with fewer logistical and resource limitations compared to monkeys. While the allometric scaling of human PK from rat, mouse, or FcRn transgenic mouse models has been explored, there are limited systematic studies in rat [7,8,12].

With the expansion of mAb therapeutic indications, the SC route of administration has gained preference due to its suitability for self-administration [9]. Following mAb injection in the SC space, a mAb must transit through the SC environment, lymphatic vessels, and lymphoid tissue via interstitial and lymph fluid flow before reaching systemic circulation [13]. Pre-systemic clearance during transit from the site of injection to circulation results in incomplete bioavailability, which in humans exhibits substantial variability among mAbs [14]. Factors such as interaction with the complex meshwork of proteins and extracellular matrix components in tissues, catabolism by local proteolytic enzymes, or even aggregation in the SC space are possible mechanistic reasons for incomplete bioavailability [15]. While in silico, in vitro, and in vivo approaches have demonstrated utility for antibody screening and clinical CL predictions, the accurate prediction of mAb bioavailability in humans has been more challenging. In 2018, the SC Drug Delivery and Development Consortium convened industry experts addressing key issues and knowledge gaps in SC delivery of biotherapeutics [16]. The prediction of SC bioavailability was identified as a key, high-priority knowledge gap increasing risk in preclinical to clinical translation of mAb PK.

To date, non-clinical in vivo models have shown limited correlation with human SC bioavailability. Richter and Jacobsen summarized IV and SC PK data for marketed immunoglobulins (IgGs) and IgG Fc fusion proteins in human, monkey, marmoset, mice, rats, and minipigs [17]. Out of the 12 molecules with human SC bioavailability data, only 2 included rat data while 10 included non-human primate (NHP) studies, which tended to overpredict human bioavailability. For the one Fc fusion protein molecule with both rat and monkey data (Rilonacept), rat was a better predictor of the 43% SC bioavailability observed in humans, with rat demonstrating 54% and monkey 70%. The reasons for the disconnect between human and non-clinical species SC bioavailability are unclear, but factors that may influence differential SC bioavailability among species include the structure of the skin, the subcutaneous proteolytic milieu, species-dependent FcRn affinity, adiposity, dose concentration, volume, total protein mass, and delivery device [13,16]. Further, for many preclinical species including rats, the correlation to human SC bioavailability has not been well studied.

In the present study, we expanded on the reported CL correlations demonstrated between human and rat [12]. In addition, we investigated cross-species bioavailability in rats, monkeys, and humans. Our mAb PK dataset was developed by mining available external and internal data. We further expanded the dataset by performing IV and SC PK studies in rats with 14 mAbs for which monkey and human data were available. Together, we obtained PK data from 25 human mAbs. Our results substantiated previous reports that human mAb CL is well predicted by both monkeys and rats and confirmed that the monkey model is a poor predictor of human bioavailability. Importantly, this analysis identified rats as the first preclinical species to demonstrate significant correlation with human mAb bioavailability. These findings support the rat as a highly valuable model for early triaging of molecules in therapeutic antibody discovery, with favorable accessibility, cost, and logistics, along with predictive power for both CL and bioavailability.

## 2. Materials and Methods

### 2.1. Non-Clinical and Clinical Data Mining

The scope of data collection was confined to humanized and fully human mAbs. Both non-clinical and clinical PK parameters were gathered from internal reports and public sources, including publications and regulatory documents. The CL and SC bioavailability parameters collected through data mining encompassed various dosing parameters (dose, dose volume, formulation concentration, site of administration) across different studies and species.

Given the variability in PK parameters reported for different mAbs due to factors such as intrinsic PK properties, immunogenicity, patient population, study design, dosing regimen, bioanalysis, data analysis methodology, and results presentation, the following guidelines were used to identify and curate representative non-specific CL and SC bioavailability values for each mAb:

#### 2.1.1. Clinical PK Parameter Collection

Population PK Model Parameters: Priority was given to collecting population estimates of non-specific CL and SC bioavailability from non-linear mixed effects models as these model parameters account for sources of PK variability and non-linearity.

Clinical Non-Compartmental Analysis (NCA) Parameter Collection: If population PK model parameters were unavailable, mean parameters were collected from NCA of single-ascending dose (SAD) studies. For each mAb, estimates of CL and/or apparent CL following SC administration (CL/F) across SAD study cohorts were evaluated for dose dependency. For mAbs exhibiting dose-dependent PK, parameter values were selected from the highest dose level explored, provided dose-linear PK was achieved in that range. For mAbs with dose-linear PK, parameter values were also selected from the highest dose level explored.

#### 2.1.2. Non-Clinical Parameter Collection

Non-clinical parameters were collected from NCA of single-dose PK experiments using the same methodology described for clinical NCA parameters. To minimize the impact of small sample sizes in non-clinical studies on identifying representative parameters, grand mean values were calculated across dose levels/cohorts deemed to represent dose-linear PK.

Typical total body weights for rats (0.3 kg), cynomolgus monkeys (3 kg), and humans (70 kg) were assumed to convert CL between weight-normalized and non-weight-normalized standardized units.

### 2.2. Animal Studies

Fourteen mAbs were identified for which human and monkey PK data were available, but rat PK data were not available. For these mAbs, rat PK studies were conducted at Labcorp (Greenfield, IN, USA). The study procedures complied with the Animal Welfare Act Regulations (Title 9 Code of Federal Regulations—Part 3) and were approved by the Institutional Animal Care and Use Committee (IACUC) of Labcorp, Inc. Sprague Dawley rats (N = 3 per route) received 3 mg/kg mAb formulated at 2 mg/mL by the IV and SC route. Formulation buffers differed among the 14 molecules tested. IV doses were administered in the lateral tail vein, whereas SC doses were administered in the dorsal thoracic region. This SC location was chosen to match past studies conducted by our group. Whole blood (0.3 mL) was collected pre-dose and at 1, 6, 12, 24, 48, 72, 96, 120, 144, 168, 240, 336, 504, 672, 840, and 1008 h post-dose and processed as serum. Serum samples were split for use in assays to determine serum concentrations of test mAbs and anti-drug antibody (ADA) response.

### 2.3. Bioanalysis

Antibody concentrations were measured from rat serum samples at Charles River Laboratories (Montreal, QC, Canada) using a qualified total human IgG enzyme-linked immunosorbent assay (ELISA). Standard curves and controls were generated for each mAb, with a curve range of 10–500 ng/mL. Wash steps were performed with phosphate buffered saline (PBS), pH 7.4 containing 0.05% Tween 20 detergent. Incubation steps were performed for 1 hour (h) with shaking at 200 revolutions per minute (rpm) unless otherwise specified. The 96-well 4 HBX ELISA plates (ThermoFisher, Waltham, MA, USA, catalog 3855) were coated with 1.0 µg/mL Goat Anti-Human Kappa (Southern Biotech, Birmingham, AL, USA, catalog 2061-01) in 0.2 M carbonate–bicarbonate buffer. Assay plates were washed, blocked with 1% casein in PBS (ThermoFisher, catalog 37528), and samples (diluted as needed with rat serum), standards, and controls were transferred to coated ELISA plates and incubated for 1 h at room temperature (RT) followed by washing. Detection of bound antibody was achieved with mouse anti-Human IgG Fc-HRP (Fcγ fragment specific, conjugated to horseradish peroxidase; Southern Biotech, Birmingham, AL, USA, catalog 9040-05) diluted 1:40,000 in assay buffer. Signal was developed with 3,3′,5,5′-tetramethylbenzidine (TMB) solution (equal parts of TMB Peroxidase Substrate and Peroxidase Substrate Solution B, Sera Care, Milford, MA, USA, catalog 5120-0052 and 5120-0039). Development was stopped with TMB Stop Solution (Sera Care, Milford, MA, USA, catalog 5150-0021), and absorbance values were read on a plate reader (Molecular Devices, Sunnyvale, CA, USA, SpectraMax M3, M5, or M2e) at 450 nm with correction at 650 nm. Unknown concentrations were determined through interpolation from the standard curve fit using a 4 or 5-parameter logistic algorithm.

Anti-drug antibodies in rat serum samples were detected using an acid-disassociation assay approach. All wash steps involved four wash cycles with tris-buffered saline containing 0.05% Tween 20 detergent. All incubations were performed on a plate shaker rotating at approximately 200 rpm. Unbound sites on the 96-well streptavidin capture (SA-C) plates (ThermoFisher, Waltham, MA, USA, catalog 15500) and 96-well MSD streptavidin assay (SA-A) plates (Meso Scale Discovery, Rockville, MD, USA, catalog L15SA) were blocked with casein buffer (ThermoFisher, Waltham, MA, USA, catalog 37528). After blocking, SA-C plate received 100 µL/well of biotin-conjugated capture antibody (5 µg/mL), and SA-A plate received 50 µL/well of biotin-conjugated capture antibody. SA-C and SA-A plates were incubated at RT for 1 h. On a third non-binding 96-well mix plate, 10 µL of sample was added to 90 µL of 300 mM acetic acid. The mix plate was incubated at RT for 5 min. SA-C was washed, and 120 µL of 1 M Tris, pH 9.0 (ThermoFisher, Waltham, MA, USA, catalog, J62084-K2), followed by 40 µL of acidified sample from the mix plate was added to each well in duplicate and incubated for 1 h. After washing, bound material was eluted from SA-C with 70 µL of 300 mM acetic acid for 5 min at RT with shaking. Prior to elution of SA-C, the SA-A plate was washed, and 150 µL of 1 M Tris pH 9.0 was added. A volume of 50 µL from SA-C was transferred to SA-A, followed by incubation for 1 h at RT. Detection antibody (goat anti-rat IgG, Meso Scale Discovery, Rockville, MD, USA, catalog R32AH-1) was diluted at 1:10,000 in blocking buffer. After a 1 h incubation and washing, signal was developed with 1× Read Buffer (Meso Scale Discovery, Rockville, MD, USA, catalog R92TC-1), and plates were read using an MSD plate reader.

### 2.4. Data Analysis and Modelling

#### 2.4.1. Rata PK Data Analysis

For the 14 rat PK studies performed to augment the rat dataset, the mAb concentration versus time profiles were visually inspected, and data points with decreased exposure consistent with ADA were omitted. The emergence of ADA in the impacted animals was confirmed with the ADA analysis. NCA was conducted in Watson LIMS (Lab Information Management System) version 7.6 (ThermoFisher Scientific, Waltham, MA, USA) to calculate CL and bioavailability. The SC bioavailability was calculated as (mean SC AUC_0–∞/_mean IV AUC_0–∞_) × 100, where AUC denotes area under the curve.

#### 2.4.2. Correlation Analysis

Spearman’s rho and simple linear regression were conducted to explore correlation among monkey, rat, and human CL and SC bioavailability using GraphPad Prism (version 9.3.1).

#### 2.4.3. Clearance Allometry Modeling

Non-linear mixed effects modeling (NLMEM) was performed to investigate the relationship between mAb CL and total body weight (BW) between pairwise species and all species (NONMEM^®^ version 7.5.0 and R version 4.1.2). The structural model assumed a classical body-weight-based allometric scaling formula expressed as follows:

(1) $$CL=\alpha \cdot {BW}^{\beta }$$

The terms CL and BW represent observed CL and assumed total body weight, respectively. The allometric coefficient (*α*) and allometric exponent (*β*) were assumed to be log-normally distributed and estimated along with inter-antibody variability (*η_i_*), which was assumed to be normally distributed with mean zero and variance ω^2^:

(2) $$\theta_{i}=\theta_{TV}\cdot e^{\eta_{i}}$$

Here, θ_i_ represents the parameter estimate for antibody *i*, θ_TV_ represents the typical parameter estimate for all antibodies, and *η_i_* is the estimate of the random effect *η* for antibody *i*. Residual unexplained variability was described by a proportional error model. Parameter estimation used the first-order conditional estimation with interaction algorithm. Model evaluation and selection were guided by the following criteria: visual inspection of model fit, inspection of residuals, convergence of the estimation and covariance routines, reasonable parameter and error estimates, and a decrease in the objective function values (OFV) between hierarchical models of at least 3.841 (α = 0.05, degree of freedom = 1). Bootstrap analysis was conducted with Perl Speaks NONMEM (version 5.3.0).

Human CL for antibody *i* was scaled from the CL determined in a single non-clinical species using a fixed allometric exponent (*β*) and BW expressed as follows:

(3) $${CL}_{i,human}={CL}_{i,non-clinical}{\left(\frac{{BW}_{human}}{{BW}_{non-clinical}}\right)}^{\beta }$$

## 3. Results

Table 1 summarizes the CL and SC bioavailability information collected by data mining or from de novo rat PK studies (rat study data are further detailed in Table A1).

**Figure A1:**
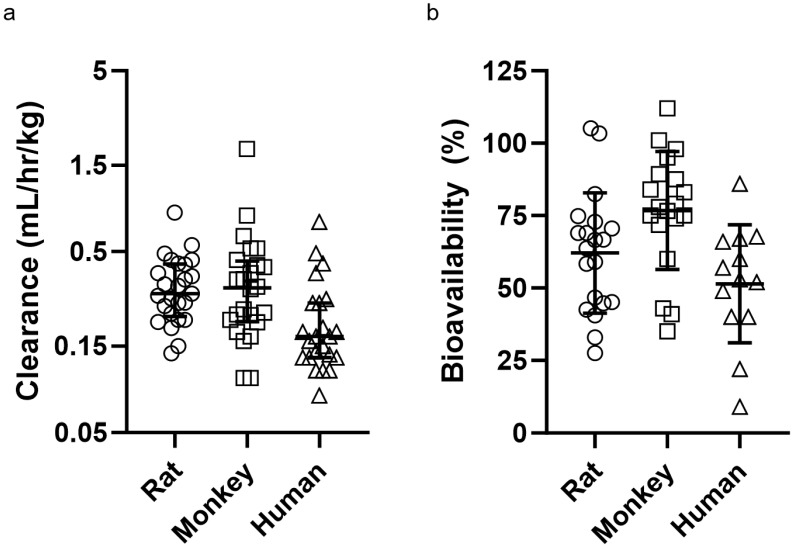
(**a**) Weight-normalized clearance for all mAbs in the dataset across species. (**b**) Subcutaneous bioavailability (%) for all mAbs in the dataset across species. Data are presented as mean ± standard deviation.

For this study, we chose human mAbs that had a wide range of clearance and SC bioavailability values to capture molecules with diverse PK characteristics. Across mAbs, rat mean CL was 0.332 mL/h/kg with a range of 0.137–0.823 mL/h/kg, monkey mean CL was 0.389 mL/h/kg with a range of 0.100–1.85 mL/h/kg, and human mean CL was 0.222 mL/h/kg with a range of 0.080–0.730 mL/h/kg (Figure A1). Across mAbs, rat mean percent bioavailability values were 62.1% with a range of 27.5–105.2%, monkey mean bioavailability values were 76.8% with a range of 35–112%, and human mean bioavailability values were 51.5% with a range of 9–86% (Figure A1).

### 3.1. Rat and Monkey Predict Human Clearance

**Figure 1:**
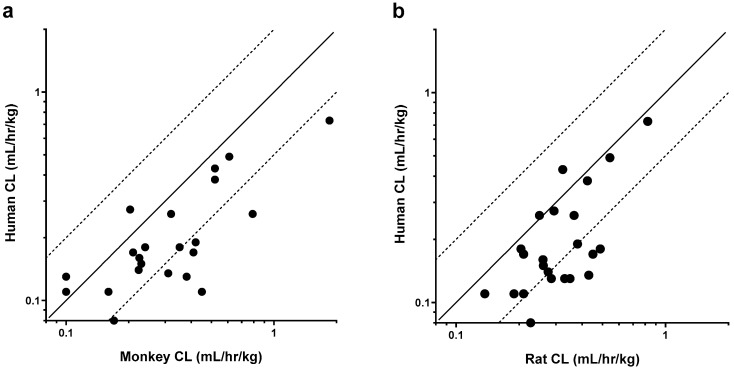
Weight-normalized clearance (CL) relationships between humans and pre-clinical species. (**a**) CL in humans versus CL in monkeys for 22 mAbs; (**b**) CL in humans versus CL in rats for 23 mAbs. Solid black lines represent the unity line, while dashed lines represent 0.5 and 2-fold change from the unity line.

Clearance correlations are depicted graphically in Figure 1, and the statistical analysis is summarized in Table 2. Weight-normalized CL was typically faster in the non-clinical species as compared to humans. As shown in Table 2, *p*-values obtained for mAb CL correlations were found to be statistically significant when comparing both monkey to human (Spearman rho = 0.67; *p*-value = 0.001) or rat to human (Spearman rho = 0.57; *p*-value = 0.004). As expected, based on the other correlations, significant association was also observed between rat and monkey CL (Spearman rho = 0.55; *p*-value = 0.005).

Parameter estimates for the final allometric CL models are provided in Table 3. The inclusion of inter-antibody variability in the allometric coefficient (ω^2^(*α*)) significantly improved the model fit across all models evaluated.

**Figure 2:**
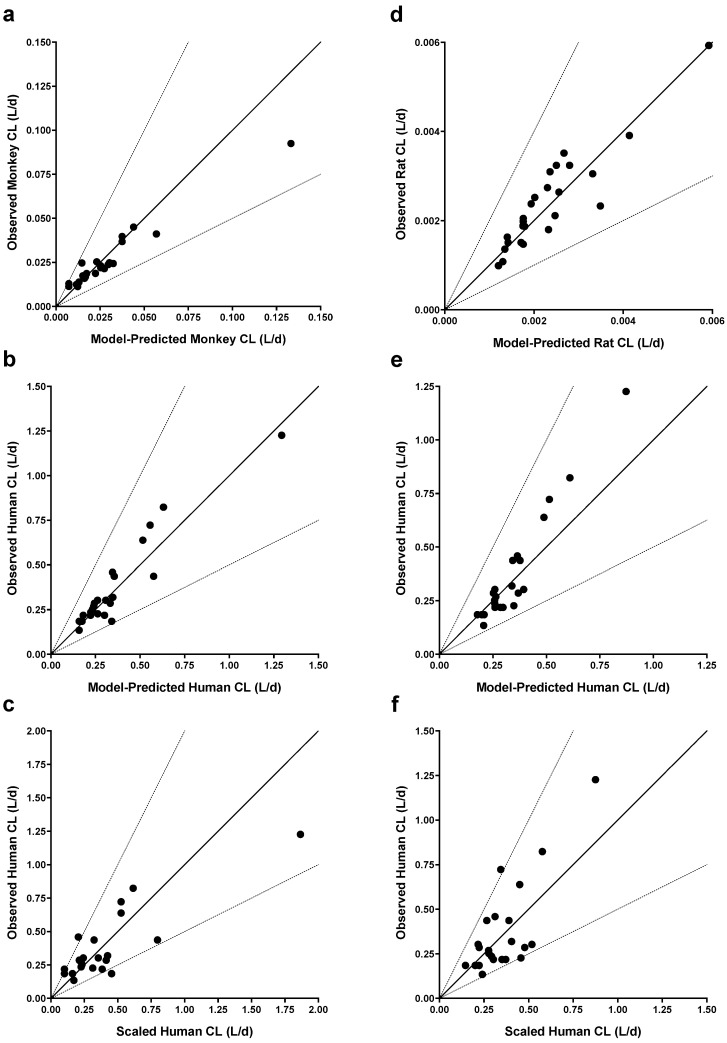
Goodness-of-fit plots and scaled human clearance (CL) for monkey-to-human (**a**–**c**) and rat-to-human (**d**–**f**) allometric CL models. Observed versus predicted CL in monkeys (**a**) and humans (**b**) from the monkey-to-human model. (**c**) Observed human CL versus human CL scaled from monkeys using an allometric exponent of 0.84. Observed versus predicted CL in rats (**d**) and humans (**e**) from the rat-to-human model. (**f**) Observed human CL versus human CL scaled from rats using an allometric exponent of 0.92. Solid lines represent the unity line, while dashed lines represent 2-fold change in slope from the unity line.

Conversely, variability in CL was not explained by including terms to describe inter-antibody variability in the allometric exponent (ω^2^(*β*)). Model parameters were estimated with good precision; relative standard errors were less than 15% for fixed-effects parameters and less than 42% for random effects. When comparing final models across each group of species evaluated, allometric coefficients (*α*_TV_) were not significantly different (Table 3). In contrast, there were notable trends in allometric exponents (*β*_TV_) across models. Scaling human CL from a single non-clinical species, *β*_TV_ was larger when scaling from rats (0.92) than monkeys (0.84), although the difference was not significant due to overlapping 95% confidence intervals (CI). For the final monkey–human (Figure 2a,b) and rat–human (Figure 2d,e) two-species models, model-predicted CL was within 2-fold of observed CL. There were no clear trends or bias in residuals versus model predicted CL for these models, although the rat–human model trended towards a slight underprediction of antibodies with the fastest human CL (Figure 2e). Scaling human CL from monkeys using an allometric exponent of 0.84 (Table 3) resulted in 19 of 22 scaled human CL values within 2-fold of observed CL (86%) and a mean observed/scaled ratio of 1.12 (data shown in Figure 2c). Similarly, scaling human CL from rats using an allometric exponent of 0.92 (Table 3) resulted in 21 of 23 scaled human CL values within 2-fold of observed CL (91%) and a mean observed/scaled ratio of 1.04 (data shown in Figure 2f).

The *β*_TV_ parameter for scaling monkey CL from rats was larger than the monkey–human and rat–human models and significantly larger than the monkey–human model based on the 95% CI. Modeling mAb CL across all three species (the rat–monkey–human model) resulted in a *β*_TV_ estimate of 0.90 between that of the monkey–human and rat–human models (Table 3). Given the similarity between *β*_TV_ estimates in the rat–human and rat–monkey–human models, predicting human CL from rat CL resulted in nearly identical results. However, human CL was generally overpredicted from monkey CL using the three-species model versus the two-species monkey–human model.

Given the limited CL data available for this analysis, we elected to include all data for model development and parameter estimation. To evaluate the robustness of parameter estimates to the underlying data, we conducted bootstrap analysis of the final allometry models using 2000 resampled datasets. Means and standard errors of bootstrapped parameter distributions were nearly identical to original model estimates; therefore, conclusions drawn from original model parameters were robust.

### 3.2. Rat SC Bioavailability Significantly Correlates with Human

**Figure 3:**
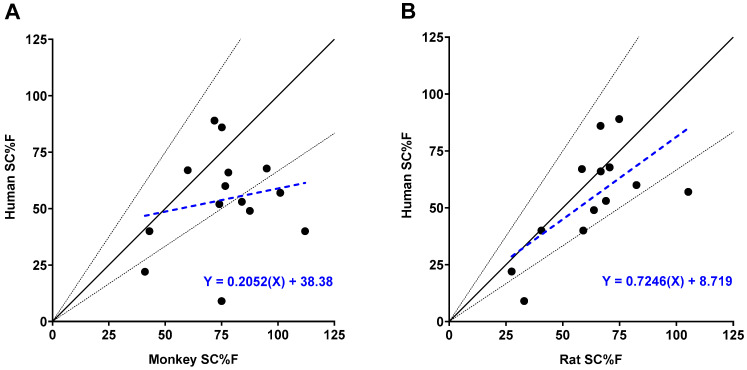
Subcutaneous bioavailability (SC%F) relationships between humans and pre-clinical species. (**A**) SC%F in humans versus SC%F in monkeys for 14 mAbs. (**B**) SC%F in humans versus SC%F in rats for 13 mAbs. Solid black lines represent the unity line, while dashed black lines represent a 1.5-fold change in slope from the unity line. Dashed blue lines and equations represent linear regression of the data points.

Among the 25 mAbs in our dataset, 14 had bioavailability data for both monkeys and humans, and 13 had bioavailability data for both rats and humans (Table 1). Generally, bioavailability in monkeys was higher than in humans, with 11 out of 14 monkey values exceeding human bioavailability. Six of these values were more than 1.5 times higher (Figure 3A, Table 1, Figure A1). Statistical analysis in Table 4 showed no significant correlation in bioavailability between monkeys and humans (Spearman rho *p* = 0.75; regression *p* = 0.53).

Similarly, bioavailability in rats (8 of 13) was generally higher than in humans (Figure 3B, Table 1, Figure A1). However, only two rat bioavailability values demonstrated rat to human ratios that were greater than 1.5-fold. In contrast to monkeys, bioavailability in rats demonstrated a positive and significant correlation with bioavailability in humans using two different statistical tests (Spearman rho *p*-value = 0.02; regression *p*-value = 0.02). In this analysis, non-clinical bioavailability values were used as experimentally determined including two values that were greater than 100%. Censoring the upper bound of non-clinical bioavailability values at 100% did not meaningfully alter these correlations.

**Figure 4:**
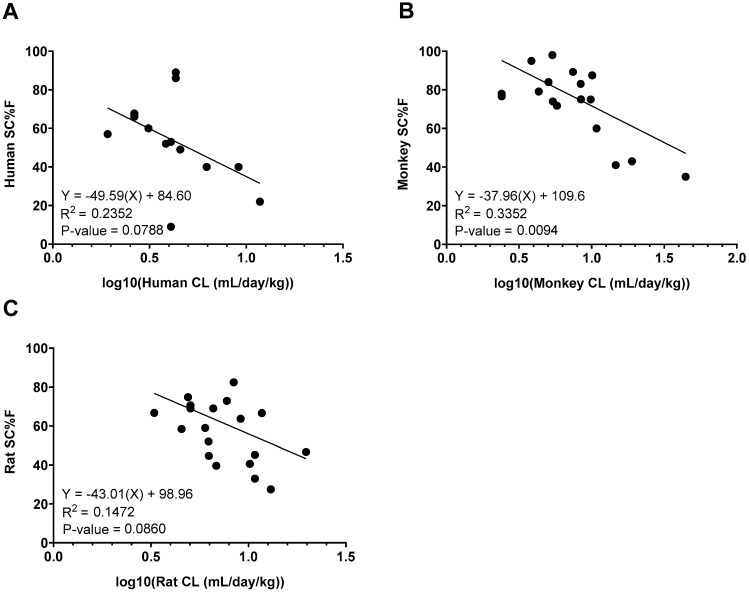
Correlation analysis between weight-normalized clearance (CL) and subcutaneous bioavailability (SC%F) in (**A**) humans, (**B**) monkeys, and (**C**) rats. Linear regression lines and analyses are shown on each graph.

Relationships between CL and SC bioavailability were also explored (Figure 4). A moderate negative correlation between CL and bioavailability was observed in monkeys (R-squared value: 0.3352), which was stronger than the relationship in humans (R-squared value: 0.2352) or rats (R-squared value: 0.1472). The correlation was only statistically significant in monkeys (*p*-value = 0.0094) while trending toward significance in humans and rats (*p*-value = 0.0788 and *p*-value = 0.0860, respectively).

## 4. Discussion

Monoclonal antibodies are an important therapeutic modality for multiple disease states [19]. Selecting antibodies with optimal absorption, distribution, metabolism, and excretion properties for clinical development increases the likelihood of achieving several important development objectives: minimizing dose, removing barriers to treatment, and improving patient experience [20]. The challenge has arisen to develop non-clinical tools that effectively aid in selecting therapeutic candidates with preferred PK properties in humans, particularly slow CL and high bioavailability. While some in silico and in vitro tools are useful in drug discovery to prioritize molecules for further engineering and optimization, in vivo PK studies remain important [5,21,22]. Further, human PK/PD projections continue to be reliant on in vivo PK data to guide early clinical development of a novel mAb.

For the purposes of this study, we focused on PK properties that are not influenced by antibody interactions with target ligand, which may result in dose or concentration-dependent disposition. These considerations were most important when analyzing PK in monkeys and humans, given that most antibodies in our study were engineered to bind to antigens in both species with high affinity. Representative non-specific PK parameter values in monkeys and humans were either selected from population-based PK models accounting for potential non-linear PK or from the NCA of single-dose PK studies at doses high enough to saturate and minimize the potential influence of target-mediated disposition due to ligand interactions. PK in rats was assumed to be driven by ligand-independent or non-specific processes given that human antibodies in our study do not cross-react to orthologous rat ligands. Across the curated studies, mAb doses were not in a range to saturate FcRn binding. Taken together, PK properties evaluated in this study depended on mechanisms or interactions governing non-specific PK properties, including the recycling activity of the FcRn receptor [11].

Humanized or fully human mAbs are foreign antigens to non-human species with the potential to elicit the formation of ADA [23]. The development of ADA can impact PK assessment through several mechanisms, such as altering the disposition of the mAb (clearing ADA) or interfering with the bioanalytical measurement of the molecule. For mAbs with slow CL and long half-lives, the appearance of clearing ADA with high reactivity and high in-study incidence limits the duration of reliable drug measurements, leading to an inadequate characterization of the AUC and increased uncertainty in determining the underlying PK properties. While a moderate incidence of ADA was observed in our de novo rat PK studies, those rats that did develop ADA had low-to-moderate reactivity, which appeared late in the sampling time course and had variable impact on serum PK as some rat mAb exposures were unaffected by ADA. There were sufficient data points that were not impacted by ADA to enable less than 25% extrapolation of AUC_0–inf_ for all individual animal results included in the dataset (Table A1). The median %CVs for AUC_inf_ were as follows: IV median 9.4% (min: 1.2%, max: 32%); SC median 18.6% (min: 2.2%, max: 35.5%). Thus, while ADA did develop in some of the rat studies, the extent of ADA did not meaningfully confound CL and SC%F estimates.

### 4.1. Rat and Monkey Are Predictive Models for mAb Clearance

Although the monkey model is considered the gold standard for the prediction of human CL, factors such as high cost, animal scarcity, and ethical considerations necessitate the development of alternative approaches. Compared to monkeys, fewer investigations have evaluated rodents for predicting antibody PK in humans. A recent publication explored various fixed allometric exponents for the single-species scaling of human CL for 28 mAbs from mice, rats, or monkeys [12]. This study showed an allometric exponent of 0.85 resulted in <2-fold prediction error for 78%, 95%, and 92% of mAbs studied for mouse, rat, and monkey, respectively. Similarly, our study demonstrated significant CL correlation between rats, monkeys, and humans (Table 2). Among the 25 antibodies in our study, the optimal allometric exponent for scaling human CL from monkeys was 0.84 with a 95% CI of 0.78–0.90, which encompasses most previously reported results [7,8,12,24]. Scaling CL from rats to humans resulted in a higher exponent of 0.92 (95% CI of 0.88–0.95) (Table 3). A model including all species resulted in a higher allometric exponent than single-species scaling from monkey but lower than that of single-species scaling from rat (Table 3). While attractive in its simplicity, if applied to monkey CL data alone, the exponent of 0.90 from the three-species model led to an over-prediction of human CL relative to the monkey-specific exponent of 0.84.

Based on 95% CIs among these models (Table 3), we cannot conclusively determine that allometric exponents for scaling mAb CL to humans should be species-specific. However, there are fundamental differences in human IgG (hIgG) affinity to the FcRn of rodents, monkeys, and humans that may influence relative CL between species. Compared to the interactions with human FcRn at acidic pH, hIgGs (IgG1, IgG2, IgG3, and IgG4 subtypes) are known to have higher affinity to FcRn expressed by mice, rats, and monkeys but retain weak affinity binding to all species FcRn at neutral pH [25]. Antibody engineering strategies to increase affinity of hIgG to human or monkey FcRn at acidic pH, with no measurable change at neutral pH, have been shown to reduce antibody CL [26,27,28]. Therefore, compared to monkeys, the clearance of wild-type hIgGs in rodents may be influenced by unique hIgG:FcRn interactions, resulting in a different relationship to human CL. Wild-type mouse FcRn affinity to hIgG has a similar pattern of pH-dependent affinity as rat FcRn, and a best-fit allometric exponent of 0.89 has been reported for predicting human CL from wild-type mice, which is similar to our best-fit exponent from rats [25,29,30].

It should be noted that mAbs included in this study were limited to molecules with wild-type FcRn binding motifs. Given the complex interplay between hIgG:FcRn and host IgG:FcRn interactions, caution should be taken if applying allometric principles in this study to scale and predict clinical PK of antibodies with modified FcRn interactions. For instance, a different allometric exponent for scaling CL of antibodies containing YTE or LS-containing mutations has been proposed [27]. Additional studies will be required to determine the relationship between CL of these and other non-wild-type human IgGs between rodent models and humans.

Across the allometric models explored in this study, model fit was not improved by inclusion of inter-antibody variability in the allometric exponent. This result indicates that a single allometric exponent is generally applicable across human IgGs and is consistent with similar FcRn affinity noted among human IgG isotypes [25,30]. In contrast, the incorporation of inter-antibody variability in the intercept uniformly improved model fit (Table 3). While covariates that may explain this variability were not explored in this study, antibodies with physiochemical attributes that increase non-specific interactions have been correlated with increased non-specific CL as reviewed by Datta-Mannan (2019) [31]. Taken together, the predictivity of future allometric CL models may be improved by the measurement and incorporation of antibody-specific properties.

### 4.2. Rat as a Predictive Model for mAb SC Bioavailability

As summarized by Zou [14] and Martin et al. [1], the subcutaneous bioavailability of current marketed mAbs varies from 49 to 100%, and the range of subcutaneous bioavailability for mAbs in clinical development is wider (29% to 100%). With limitations in the dose volume that can be comfortably administered as a subcutaneous bolus, the bioavailability of a mAb can have a substantial impact on the overall clinical dosing paradigm and patient experience. Consequently, the development of tools to enable the prediction of mAb bioavailability has become an urgent topic in drug discovery research.

The transit of mAbs from injection site to systemic circulation may be affected by multiple physiological factors such as differing SC tissue structures across various anatomical locations, flow rates of interstitial or lymphatic fluid, and others [32,33,34]. Factors intrinsic to an individual mAb such as isoelectric point, positive charge, or hydrophobicity have also been proposed to contribute to differences in absorption and pre-systemic clearance [9,14,15]. However, studies investigating correlation between mAb biophysical characteristics and SC bioavailability are limited and provide differing conclusions. A study by Datta-Mannan et al. [35] examined five pairs of parental and re-engineered antibodies in monkeys and suggested that the integrated interaction of multiple biophysical factors was at play in determining their bioavailability. Bender and colleagues investigated the ability of several in vitro assay formats to predict SC bioavailability and other PK parameters and found that none of the approaches were useful in predicting human bioavailability [36].

FcRn interactions have also been proposed to influence mAb SC bioavailability, though studies exploring these interactions similarly are limited. Deng and colleagues studied murinized antibodies engineered with different binding affinities for the mouse FcRn receptor [37]. PK studies in severe combined immunodeficient mice demonstrated increased bioavailability when a variant had higher FcRn affinity at pH 6.0 and unchanged affinity at 7.4. Interestingly, a mIgG2a clone with no detectable mFcRn binding at pH 6.0 was 41.8% bioavailable following SC administration. In contrast, antibody pairs analyzed by Datta-Mannan and colleagues included engineered human mAbs with T250Q/M428L mutations, which increased FcRn binding affinity in monkeys [35]. While the enhanced FcRn interactions resulted in decreased CL relative to the wild-type versions in monkeys, they did not translate to improved SC bioavailability.

Minipigs are often used as a model for humans as their skin exhibits similar epidermal and stratum corneum thickness, lipid composition, and lymph architecture [38,39,40]. One notable difference is the presence of the panniculus carnosus in pig skin, which is a layer of striated muscle within the subcutaneous tissue [41]. The panniculus carnosus is an anatomical feature that is present to a variable extent across mammalian species but is mostly absent in humans [42]. It is undetermined whether its presence influences the rate or extent of absorption of biologics administered by the SC route.

Zheng et al. evaluated minipigs for the subcutaneous (SC) bioavailability of mAbs [43]. They administered nine human mAbs via IV and SC routes to minipigs. While CL values between minipigs and humans were correlated, SC bioavailability in minipigs only showed a weak correlation with human values for six of the mAbs. The binding affinities (K_D_ at pH 6) of all nine mAbs were within two-fold when comparing minipig FcRn to human FcRn. This suggests that factors beyond CL, FcRn interactions, and common skin features contribute to the lack of correlation in bioavailability between humans and minipigs. Similarly, human wild-type IgG affinity to monkey FcRn at acidic pH (5.8–6) is within 2–3-fold of human FcRn affinity [25,43]. Despite this, SC bioavailability does not significantly correlate between monkeys and humans.

In contrast, rat skin is generally thinner than human skin, with only about 10 to 15% of the combined dermal and epidermal thickness [44]. Rat skin also includes the panniculus carnosus muscle layer, located below the dermal white adipose and above the interstitial connective tissue overlying the fascia [42]. Additionally, at pH 5.8, human IgG binds to rat FcRn with an approximately 10-fold lower K_D_ compared to human FcRn [25]. Despite these differences, a significant correlation was observed between rat and human bioavailability for the mAbs studied. These findings suggest that there may be undefined interactions within the SC space or lymphatic system common to both rats and humans that strongly influence mAb bioavailability following SC administration.

The injection (or administration) site is a factor that merits further investigation. The dorsal interscapular region, which is a common site of administration in non-clinical studies, was used for subcutaneous injection of the 14 antibodies investigated in the de novo rat studies presented in this paper. In humans, abdomen, upper arm, and thigh are commonly used sites for SC dosing [32]. A survey of clinical PK data from the literature and the US Food and Drug Administration (FDA) database showed that among the 19 antibodies, 5 exhibited site-dependent PK when comparing the abdomen and thigh or abdomen and arm [32]. These antibodies typically had a half-life of less than 15 days or a T_max_ of less than 5 days. Generally, bioavailability was highest following injection in the thigh, with the abdomen showing similar or slightly higher bioavailability compared to the arm. Zou et al. noted that this rank order matched the average lymph flow in these regions [32]. The fact that only a subset of mAbs showed injection-site-specific PK suggests that mAb-specific attributes influence differential bioavailability.

Studies on the SC bioavailability of rituximab in rats and mice indicated that injection site, volume, and total dose significantly affect bioavailability [45,46]. Our observations, showing a correlation between rat and human bioavailability, support the need for further studies to determine if the rat model can elucidate injection location-mediated differences in bioavailability.

Finally, our data demonstrated inverse correlations between CL and bioavailability in rats, monkeys, and humans, with statistical significance in monkeys. Other groups have also reported a correlation between CL and bioavailability, though only for clinical data [14,47]. This consistent association across species suggests that optimizing properties to reduce CL could improve bioavailability. Further studies are needed to gain more insight into this relationship.

## 5. Conclusions

The findings in this report offer valuable tools for therapeutic antibody discovery. Using the rat model allows for early PK assessment, helping to distinguish molecules in the discovery pipeline that might have ambiguous CL properties based on in silico or in vitro characteristics. Rat PK data can also be used to predict human CL following SC administration, which is the preferred dosing route for many mAb therapeutics. Our data show an inverse correlation between CL and bioavailability, suggesting that optimizing mAb CL characteristics early on may also improve bioavailability. To our knowledge, this is the first systematic evaluation of mAb SC bioavailability across rats, monkeys, and humans. The rat model offers clear cost and availability advantages over non-human primates or transgenic mice, making drug development more agile and cost-effective.

## Figures and Tables

**Table 1:** Weight-normalized mAb clearance (CL) and subcutaneous bioavailability (SC%F).

Monoclonal Antibody	Rat CL (mL/h/kg)	Monkey CL (mL/h/kg)	Human CL (mL/h/kg)	Rat SC%F	Monkey SC%F	Human SC%F
alirocumab	0.488 ^a^	0.352 ^c^	0.180 ^b^	66.6 ^a^	75.1	86.0 ^b^
canakinumab	0.189 ^a^	0.450 ^c^	0.110 ^b^	58.4 ^a^	60.0	67.0 ^b^
guselkumab	0.380 ^a^	0.420 ^c^	0.190 ^b^	63.7 ^a^	87.5	49.0 ^b^
secukinumab	0.137 ^a^	0.100 ^c^	0.110 ^b^	66.7 ^a^	78.0	66.0 ^b^
tabalumab	0.227 ^a^	0.170 ^c^	0.080 ^c^	105.2 ^a^	101.0	57.0 ^b^
ustekinumab	0.210 ^a^	0.160 ^c^	0.110 ^b^	70.6 ^a^	95.0	67.8 ^b^
bevacizumab	0.275 ^c^	0.223 ^c^	0.140 ^b^	69.0	98.0	-
ocrelizumab	0.330 ^c^	-	0.130 ^b^	-	-	-
risankizumab	0.204 ^a^	0.240 ^c^	0.180 ^b^	74.8 ^a^	71.8	89.0 ^b^
mAb 1	0.210 ^c^	0.210	0.170 ^c^	69.0	84.0	53.0 ^c^
mAb 2	0.260	0.225	0.160 ^c^	-	74.0	52.0 ^c^
mAb 3	0.250 ^c^	0.790 ^c^	0.260 ^c^	59.0 ^c^	43.0 ^c^	40.0 ^c^
mAb 4	0.366 ^a^	0.320 ^c^	0.260 ^c^	103.4 ^a^	-	-
mAb 5	0.261 ^a^	0.230 ^c^	0.150 ^c^	44.7 ^a^	-	-
mAb 6	0.285 ^a^	0.380 ^c^	0.130 ^c^	42.5 ^a^	-	-
mAb 7	0.350 ^c^	0.100 ^c^	0.130 ^c^	82.4 ^c^	76.6 ^c^	60.0 ^c^
mAb 8	0.430 ^c^	0.310 ^c^	0.135 ^c^	-	89.3 ^c^	-
mAb 9	0.543 ^a^	0.610 ^c^	0.490 ^c^	27.5 ^a^	41.0 ^c^	22.0 ^c^
mAb 10	0.450 ^c^	0.410 ^c^	0.170 ^c^	33.0 ^c^	75.0 ^c^	9.0 ^c^
mAb 11	0.293 ^c^	0.203 ^c^	0.273 ^c^	-	-	-
mAb 12	0.823 ^a^	1.850 ^c^	0.730 ^c^	46.7 ^a^	35.0 ^c^	-
mAb 13	0.323 ^a^	0.520 ^c^	0.430 ^c^	72.9 ^a^	-	-
mAb 14	0.424 ^a^	0.520 ^c^	0.380 ^c^	40.6 ^a^	112.0 ^c^	40.0 ^c^
mAb 15	0.450 ^c^	0.350 ^c^	-	45.2 ^c^	83.1 ^c^	-
mAb 16	0.150 ^c^	0.180 ^c^	-	-	79.1 ^c^	-

^a^ Rat PK data generated in this study (Table A1); ^b^ FDA label (Drugs@FDA: FDA-approved drugs) [18]; ^c^ internal unpublished data mL/h/kg, milliliter per hour per kilogram.

**Table 2:** Correlation analysis between rat, monkey, and human weight-normalized clearance (CL).

Spearman rho	Monkey CL vs. Human CL ^a^	Rat CL vs. Human CL ^a^	Rat CL vs. Monkey CL ^a^
R	0.67	0.57	0.55
95% CI	0.33 to 0.85	0.20 to 0.80	0.18 to 0.79
*p*-value	0.001	0.004	0.005
Sample size	22	23	24

^a^ Clearance values used in the comparison were in units of mL/h/kg, milliliter per hour per kilogram. R, Spearman correlation coefficient; 95% CI, 95% confidence interval.

**Table 3:** Allometric clearance (CL) models.

Parameters	Monkey-to-Human	Rat-to-Human	Rat-to-Monkey	3-Species
*α*_TV_ ^a^ (%RSE)	0.0087 (15)	0.0065 (8)	0.0074 (10)	0.0072 (9)
95% CI	0.0062–0.0112	0.0055–0.0075	0.006–0.0088	0.0059–0.0085
*β*_TV_ ^b^ (%RSE)	0.84 (4)	0.92 (2)	1.02 (4)	0.90 (2)
95% CI	0.78–0.90	0.88–0.95	0.94–1.10	0.87–0.94
ω^2^(*α*) ^c^ (%RSE)	0.27 (38)	0.17 (35)	0.22 (42)	0.23 (40)
σ^2 d^ (%RSE)	0.08 (23)	0.07 (18)	0.08 (33)	0.1 (23)

^a^ Typical value of allometric coefficient; ^b^ typical value of allometric exponent; ^c^ inter-antibody variance in allometric coefficient; ^d^ residual variance. 95% CI, 95% confidence interval; %RSE, percent relative standard error.

**Table 4:** Correlation analysis between rat, monkey, and human subcutaneous bioavailability (SC%F).

Correlation Coefficient	Monkey SC%F vs. Human SC%F	Rat SC%F vs. Human SC%F	Rat SC%F vs. Monkey SC%F
Spearman rho			
R	0.09	0.63	0.39
95% CI	−0.47 to 0.61	0.11 to 0.88	−0.15 to 0.75
*p*-value	0.75	0.02	0.14
Simple linear regression			
r^2^	0.03	0.43	0.16
*p*-value	0.53	0.02	0.13
Sample size	14	13	16

95% CI, 95% confidence interval; R, Spearman correlation coefficient; r^2^, coefficient of determination.

## Data Availability

Data can be made available upon request from the authors if not given in this publication.

## Appendix A

**Table A1:** Summary of newly generated (de novo) rat PK data.

Monoclonal Antibody	Animal Number	IV AUC_inf_ (µg × h/mL)	IV AUC % Extrapolated	IV T_last_ (h)	Animal Number	SC AUC_inf_ (µg × h/mL)	SC AUC % Extrapolated	SC T_last_ (h)
alirocumab	1	10,200	1.42	1008	4	6280	2.20	504
	2	9390	0.0	1008	5	8120	3.54	840
	3	9880	1.31	1008	6	5890	3.46	336
canakinumab	1	16,800	4.37	840	4	11,600	0.18	504
	2	18,000	10.5	1008	5	9070	0.54	336
	3	13,600	0.48	672	6	7590	0.01	336
guselkumab	1	6720	0.16	504	4	8000	6.00	1008
	2	12,500	8.65	1008	5	4970	0.10	336
	3	6590	0.34	504	6	3480	1.03	168
secukinumab	1	22,400	21.2	1008	4	11,000	0.47	504
	2	20,800	17	1008	5	15,100	7.32	672
	3	22,500	16.9	1008	6	17,700	18.7	672
tabalumab	1	11,400	2.61	672	4	15,800	16.2	1008
	2	14,500	11.2	1008	5	9790	0.22	504
	3	14,200	12.7	1008	6	16,700	17.10	1008
ustekinumab	1	13,300	10.2	1008	4	7420	0.24	336
	2	14,000	11.7	1008	5	10,100	1.71	1008
	3	15,700	11.2	1008	6	12,900	8.50	1008
risankizumab	1	15,100	14.3	1008	4	12,200	10.4	1008
	2	15,200	11.9	1008	5	10,800	9.85	1008
	3	13,900	12	1008	6	9970	1.29	1008
mAb 4	1	8090	1.72	336	4	7340	0.10	672
	2	8300	0.02	504	5	5500	2.00	336
	3	-	-	-	6	12,600	15.5	840
mAb 5	1	12,600	5.16	1008	4	5130	0.72	336
	2	12,400	7.31	1008	5	5380	1.90	336
	3	9930	3.23	1008	6	5140	3.50	504
mAb 6	1	11,400	15.4	1008	4	5160	15.3	336
	2	11,200	15.7	1008	5	3860	7.26	240
	3	9280	12.5	1008	6	-	-	-
mAb 9	1	4800	0.66	1008	4	1280	0.10	504
	2	5740	2.64	1008	5	1010	1.14	240
	3	6230	0.14	504	6	2320	5.72	1008
mAb 12	1	3430	0.19	504	4	2110	0.44	336
	2	4190	2.57	1008	5	1340	0.42	240
	3	3420	2.17	1008	6	1720	0.26	240
mAb 13	1	9940	5.83	1008	4	4430	0.16	336
	2	8820	4.89	1008	5	9120	6.14	1008
	3	9150	3.76	1008	6	-	-	-
mAb 14	1	6320	1.33	1008	4	3290	0.00	336
	2	6870	1.52	1008	5	2200	3.32	240
	3	8320	4.4	1008	6	3240	0.47	336

AUC, area under curve; AUC_inf_, AUC from time of dosing extrapolated to infinity; AUC % Extrapolated, percentage of AUC_inf_ due to extrapolation from T_last_ to infinity; IV, intravenous; SC, subcutaneous; T_last_, time of last measurable concentration used for data analysis.

## References

[1] Martin K.P., Grimaldi C., Grempler R., Hansel S., Kumar S. (2023). Trends in industrialization of biotherapeutics: A survey of product characteristics of 89 antibody-based biotherapeutics. mAbs.

[2] Tang Y., Li X., Cao Y. (2021). Which factors matter the most? Revisiting and dissecting antibody therapeutic doses. Drug Discov. Today.

[3] Bumbaca D., Boswell C.A., Fielder P.J., Khawli L.A. (2012). Physiochemical and biochemical factors influencing the pharmacokinetics of antibody therapeutics. AAPS J..

[4] Liu L. (2018). Pharmacokinetics of monoclonal antibodies and Fc-fusion proteins. Protein Cell.

[5] Hotzel I., Theil F.P., Bernstein L.J., Prabhu S., Deng R., Quintana L., Lutman J., Sibia R., Chan P., Bumbaca D. (2012). A strategy for risk mitigation of antibodies with fast clearance. mAbs.

[6] Kraft T.E., Richter W.F., Emrich T., Knaupp A., Schuster M., Wolfert A., Kettenberger H. (2020). Heparin chromatography as an in vitro predictor for antibody clearance rate through pinocytosis. mAbs.

[7] Ling J., Zhou H., Jiao Q., Davis H.M. (2009). Interspecies scaling of therapeutic monoclonal antibodies: Initial look. J. Clin. Pharmacol..

[8] Dong J.Q., Salinger D.H., Endres C.J., Gibbs J.P., Hsu C.P., Stouch B.J., Hurh E., Gibbs M.A. (2011). Quantitative prediction of human pharmacokinetics for monoclonal antibodies: Retrospective analysis of monkey as a single species for first-in-human prediction. Clin. Pharmacokinet..

[9] Sanchez-Felix M., Burke M., Chen H.H., Patterson C., Mittal S. (2020). Predicting bioavailability of monoclonal antibodies after subcutaneous administration: Open innovation challenge. Adv. Drug Deliv. Rev..

[10] James B.H., Papakyriacou P., Gardener M.J., Gliddon L., Weston C.J., Lalor P.F. (2021). The Contribution of Liver Sinusoidal Endothelial Cells to Clearance of Therapeutic Antibody. Front. Physiol..

[11] Ryman J.T., Meibohm B. (2017). Pharmacokinetics of Monoclonal Antibodies. CPT Pharmacomet. Syst. Pharmacol..

[12] Mahmood I. (2021). A Single Animal Species-Based Prediction of Human Clearance and First-in-Human Dose of Monoclonal Antibodies: Beyond Monkey. Antibodies.

[13] Collins D.S., Kourtis L.C., Thyagarajapuram N.R., Sirkar R., Kapur S., Harrison M.W., Bryan D.J., Jones G.B., Wright J.M. (2017). Optimizing the Bioavailability of Subcutaneously Administered Biotherapeutics Through Mechanochemical Drivers. Pharm. Res..

[14] Zou P. (2023). Predicting Human Bioavailability of Subcutaneously Administered Fusion Proteins and Monoclonal Antibodies Using Human Intravenous Clearance or Antibody Isoelectric Point. AAPS J..

[15] Richter W.F., Bhansali S.G., Morris M.E. (2012). Mechanistic determinants of biotherapeutics absorption following SC administration. AAPS J..

[16] Collins D.S., Sanchez-Felix M., Badkar A.V., Mrsny R. (2020). Accelerating the development of novel technologies and tools for the subcutaneous delivery of biotherapeutics. J. Control Release.

[17] Richter W.F., Jacobsen B. (2014). Subcutaneous absorption of biotherapeutics: Knowns and unknowns. Drug Metab. Dispos..

[18] Drugs@FDA: FDA-Approved Drugs. https://www.accessdata.fda.gov/scripts/cder/daf/index.cfm.

[19] Lu R.M., Hwang Y.C., Liu I.J., Lee C.C., Tsai H.Z., Li H.J., Wu H.C. (2020). Development of therapeutic antibodies for the treatment of diseases. J. Biomed. Sci..

[20] Wang B., Gallolu Kankanamalage S., Dong J., Liu Y. (2021). Optimization of therapeutic antibodies. Antib. Ther..

[21] Avery L.B., Wade J., Wang M., Tam A., King A., Piche-Nicholas N., Kavosi M.S., Penn S., Cirelli D., Kurz J.C. (2018). Establishing in vitro in vivo correlations to screen monoclonal antibodies for physicochemical properties related to favorable human pharmacokinetics. mAbs.

[22] Bei R., Thomas J., Kapur S., Woldeyes M., Rauk A., Robarge J., Feng J., Abbou Oucherif K. (2024). Predicting the clinical subcutaneous absorption rate constant of monoclonal antibodies using only the primary sequence: A machine learning approach. mAbs.

[23] Ponce R., Abad L., Amaravadi L., Gelzleichter T., Gore E., Green J., Gupta S., Herzyk D., Hurst C., Ivens I.A. (2009). Immunogenicity of biologically-derived therapeutics: Assessment and interpretation of nonclinical safety studies. Regul. Toxicol. Pharmacol..

[24] Betts A., Keunecke A., van Steeg T.J., van der Graaf P.H., Avery L.B., Jones H., Berkhout J. (2018). Linear pharmacokinetic parameters for monoclonal antibodies are similar within a species and across different pharmacological targets: A comparison between human, cynomolgus monkey and hFcRn Tg32 transgenic mouse using a population-modeling approach. mAbs.

[25] Abdiche Y.N., Yeung Y.A., Chaparro-Riggers J., Barman I., Strop P., Chin S.M., Pham A., Bolton G., McDonough D., Lindquist K. (2015). The neonatal Fc receptor (FcRn) binds independently to both sites of the IgG homodimer with identical affinity. mAbs.

[26] Dall’Acqua W.F., Kiener P.A., Wu H. (2006). Properties of human IgG1s engineered for enhanced binding to the neonatal Fc receptor (FcRn). J. Biol. Chem..

[27] Haraya K., Tachibana T. (2023). Translational Approach for Predicting Human Pharmacokinetics of Engineered Therapeutic Monoclonal Antibodies with Increased FcRn-Binding Mutations. BioDrugs.

[28] Robbie G.J., Criste R., Dall’acqua W.F., Jensen K., Patel N.K., Losonsky G.A., Griffin M.P. (2013). A novel investigational Fc-modified humanized monoclonal antibody, motavizumab-YTE, has an extended half-life in healthy adults. Antimicrob. Agents Chemother..

[29] Avery L.B., Wang M., Kavosi M.S., Joyce A., Kurz J.C., Fan Y.Y., Dowty M.E., Zhang M., Zhang Y., Cheng A. (2016). Utility of a human FcRn transgenic mouse model in drug discovery for early assessment and prediction of human pharmacokinetics of monoclonal antibodies. mAbs.

[30] Neuber T., Frese K., Jaehrling J., Jager S., Daubert D., Felderer K., Linnemann M., Hohne A., Kaden S., Kolln J. (2014). Characterization and screening of IgG binding to the neonatal Fc receptor. mAbs.

[31] Datta-Mannan A. (2019). Mechanisms Influencing the Pharmacokinetics and Disposition of Monoclonal Antibodies and Peptides. Drug Metab. Dispos..

[32] Zou P., Wang F., Wang J., Lu Y., Tran D., Seo S.K. (2021). Impact of injection sites on clinical pharmacokinetics of subcutaneously administered peptides and proteins. J. Control. Release.

[33] Dahlberg A.M., Kaminskas L.M., Smith A., Nicolazzo J.A., Porter C.J., Bulitta J.B., McIntosh M.P. (2014). The lymphatic system plays a major role in the intravenous and subcutaneous pharmacokinetics of trastuzumab in rats. Mol. Pharm..

[34] Kagan L., Gershkovich P., Mendelman A., Amsili S., Ezov N., Hoffman A. (2007). The role of the lymphatic system in subcutaneous absorption of macromolecules in the rat model. Eur. J. Pharm. Biopharm..

[35] Datta-Mannan A., Witcher D.R., Lu J., Wroblewski V.J. (2012). Influence of improved FcRn binding on the subcutaneous bioavailability of monoclonal antibodies in cynomolgus monkeys. mAbs.

[36] Bender C., Eichling S., Franzen L., Herzog V., Ickenstein L.M., Jere D., Nonis L., Schwach G., Stoll P., Venczel M. (2022). Evaluation of In Vitro Tools to Predict the In Vivo Absorption of Biopharmaceuticals Following Subcutaneous Administration. J. Pharm. Sci..

[37] Deng R., Meng Y.G., Hoyte K., Lutman J., Lu Y., Iyer S., DeForge L.E., Theil F.P., Fielder P.J., Prabhu S. (2012). Subcutaneous bioavailability of therapeutic antibodies as a function of FcRn binding affinity in mice. mAbs.

[38] Hammond S.A., Tsonis C., Sellins K., Rushlow K., Scharton-Kersten T., Colditz I., Glenn G.M. (2000). Transcutaneous immunization of domestic animals: Opportunities and challenges. Adv. Drug Deliv. Rev..

[39] Jacobs A. (2006). Use of nontraditional animals for evaluation of pharmaceutical products. Expert. Opin. Drug Metab. Toxicol..

[40] Sharma R., Wang W., Rasmussen J.C., Joshi A., Houston J.P., Adams K.E., Cameron A., Ke S., Kwon S., Mawad M.E. (2007). Quantitative imaging of lymph function. Am. J. Physiol. Heart Circ. Physiol..

[41] Rosh E.H., Vistnes L.M., Ksander G.A. (1977). The panniculus carnosus in the domestic pig. Plast. Reconstr. Surg..

[42] Bergman R.A., Afifi A.K., Miyauchi R. Anatomy Atlases: Illustrated Encyclopedia of Human Anatomic Variation: Opus I: Muscular System: Alphabetical Listing of Muscles: P: Panniculus Carnosus. https://www.anatomyatlases.org/AnatomicVariants/MuscularSystem/Text/P/05Panniculus.shtml.

[43] Zheng Y., Tesar D.B., Benincosa L., Birnbock H., Boswell C.A., Bumbaca D., Cowan K.J., Danilenko D.M., Daugherty A.L., Fielder P.J. (2012). Minipig as a potential translatable model for monoclonal antibody pharmacokinetics after intravenous and subcutaneous administration. mAbs.

[44] O’Brien K., Bhatia A., Tsen F., Chen M., Wong A.K., Woodley D.T., Li W. (2014). Identification of the critical therapeutic entity in secreted Hsp90alpha that promotes wound healing in newly re-standardized healthy and diabetic pig models. PLoS ONE.

[45] Kagan L., Turner M.R., Balu-Iyer S.V., Mager D.E. (2012). Subcutaneous absorption of monoclonal antibodies: Role of dose, site of injection, and injection volume on rituximab pharmacokinetics in rats. Pharm. Res..

[46] Kagan L., Zhao J., Mager D.E. (2014). Interspecies pharmacokinetic modeling of subcutaneous absorption of rituximab in mice and rats. Pharm. Res..

[47] Haraya K., Tachibana T., Nezu J. (2017). Quantitative prediction of therapeutic antibody pharmacokinetics after intravenous and subcutaneous injection in human. Drug Metab. Pharmacokinet..

